# Relationship between religiosity domains and traits from borderline and schizotypal personality disorders in a Brazilian community sample

**DOI:** 10.1590/2237-6089-2019-0085

**Published:** 2020-10-08

**Authors:** Lucas de Francisco Carvalho, Daniele Elvira Vaz Sagradim, Giselle Pianowski, André Pereira Gonçalves

**Affiliations:** 1 Universidade São Francisco CampinasSP Brazil Universidade São Francisco (USF), Campus Swift, Campinas, SP, Brazil.

**Keywords:** Personality assessment, religion, mental disorders

## Abstract

**Introduction:**

Research suggests that religiosity domains are associated with mental health constructs. Some studies have focused on the relationship between religiosity and personality disorders.

**Objective:**

To investigate the relationship between religiosity domains and pathological traits of the borderline (BPD) and schizotypal (SZPD) personality disorders.

**Methods:**

Participants were 751 adults from the general population who answered the Multidimensional Inventory for Religious/Spiritual Well-Being (MI-RSWB-E), the Attachment to God Inventory (AGI), and factors of the Dimensional Clinical Personality Inventory 2 (IDCP-2). Pearson’s correlation and regression analysis were conducted with pathological traits as independent variables and religiosity domains as dependent variables.

**Results:**

Correlation and regression analyses indicated slightly higher associations between religiosity domain and BPD traits in comparison to SZPD traits. BPD traits showed higher associations with the hope immanent, forgiveness and hope transcendent domains, while SZPD presented higher associations with connectedness. The SZPD-related paranormality factor presented the highest correlation observed in the study and was the best SZPD predictor of religiosity domains. The BPD-related hopelessness factor was the predictor with significant contribution to most regression models. BPD traits presented slightly higher average association with religiosity domains, whereas spiritual-related domains (e.g., connectedness) tended to show higher associations with SZPD traits.

**Conclusions:**

Our findings help explain the relationship between specific pathological traits and religiosity domains.

## Introduction

Associations between religiosity domains and mental health constructs have been repeatedly observed in previous studies,^1 - 7^ including those focused on the pathological traits that compose personality disorders (PD).^8 - 11^ Our aim with this study was to investigate the relationship between specific pathological traits (i.e., traits of the borderline and schizotypal PDs) and religiosity domains.

## Background

Religiosity is characterized by religious experience and faith, which impact on individual perceptions, values, daily experiences, and notions of self.^12^ Religiosity is also related to a particular ritualistic cultural component, and to a particular religion, in which there is a belief in a doctrine, attributing practices and customs of worship to a faith that is shared with a group.^13 , 14^ Although not consensual in literature,^14 , 15^ in this study we considered spirituality as one of the several domains composing the multidimensional construct religiosity.

According to Unterrainer et al.,^11^ religiosity can be grouped in two broad belief components, namely general religiosity and connectedness. General religiosity refers to institutions, traditions and religious communities. It is related to extrinsic and intrinsic religiosity, i.e., the use of religiosity for its own benefit (extrinsic), and the spiritual and religious internal experience (intrinsic).^16^ Connectedness is the deinstitutionalized expression of beliefs, related to the concept of spirituality, i.e., connectivity with a superior power or entity.

Previous studies have suggested that the level and expression of religiosity is associated with personality traits,^6 , 8 , 17^ although mixed findings have been observed regarding the direction of this relationship. Evidence of positive associations suggests religiosity to be a protective factor for personality trait expressions, while negative associations suggest religiosity to be a risk factor for the manifestation of pathological traits.

Pathological traits composing borderline personality disorder (BPD) and schizotypal personality disorder (SZPD) seem to present the strongest association with religiosity domains in comparison to other pathological traits.^8^ BPD is characterized by emotional instability, impulsivity, risk exposure, and a tendency to be hostile, including impairment of emotional expression, self-image, and interpersonal relationship.^18 , 19^ SZPD, in turn, refers to a pattern of eccentric behavior and thoughts, with an impaired ability to establish interpersonal relationship and emotional closeness, as well as a tendency to be interpersonally suspicious.^18 , 20^

Evidence points to negative associations between BPD traits and religiosity domains, specifically, religious and spiritual well-being,^19^ while religious practice and general religiosity are associated with traits such as aggressivity, mood instability, feeling of emptiness, and self-mutilating tendency.^21^ For SZPD traits, although mixed findings have been observed (e.g., Diduca & Joseph^22^ ), positive associations were found for religious attachment, while negative associations were found for religious and spiritual well-being.^4^ Moreover, positive associations between religiosity/spiritual well-being and neuroticism and magical thinking have been reported,^11^ as well as positive associations between spirituality scores (e.g., connectedness) and several SZPD traits.^9^

Even though an increase can be observed in the number of studies investigating associations between religiosity domains and pathological traits, evidence presented in previous literature is insufficient to allow more stable conclusions. Our aim in this study was to investigate the relationship between religiosity domains and pathological traits typical of BPD and SZPD. We tested two hypotheses: h1) BPD and SZPD traits should present moderate negative associations with religiosity domains, although BPD traits should show higher associations^19 , 21^ ; and h2) spiritual-related domains (i.e., connectedness and experiences of sense and meaning) should present positive moderate associations with SZPD traits, including insecure religious attachment.^4 , 9 , 11^

## Materials and methods

### Participants

Using a cross-sectional design, we recruited a non-probabilistic convenience sample comprised of 751 individuals from the general population, aged between 18 and 71 years (mean = 25.15; standard deviation = 8.37), mostly women (74.1%), self-declared white (66.6%), college students (49.4%), and single (75.5%). From the total sample, 58.9% reported having participated in psychotherapy, and 29.8% reported having received psychiatric treatment.

### Measures

#### Multidimensional Inventory for Religious/Spiritual Well-Being (MI-RSWB-E)

The MI-RSWB-E is a self-report measure used to assess religious and spiritual well-being, defined by the developers^17^ as the ability to experience and integrate meaning and purpose into existence through a connection with self, others or a higher entity. The scale is composed of six dimensions: general religiosity (8 items), forgiveness (8 items), hope immanent (8 items), connectedness (8 items), hope transcendent (8 items), and experience of sense and meaning (8 items). Psychometric properties of the scale were previously investigated.^11^ Reliability ranged between 0.70 (hope transcendent) and 0.95 (general religiosity).

#### Attachment to God Inventory (AGI)

The self-report AGI (Beck & McDonald, 2004) is designed to measure attachment with God through two dimensions: intimacy avoidance (14 items) and abandonment anxiety (14 items). Higher scores are related to insecure attachment to God. Psychometric properties were suitable in a previous study.^23^ Internal consistency for our sample was 0.53 (intimacy avoidance factor) and 0.84 (abandonment anxiety).

#### Dimensional Clinical Personality Inventory 2 (IDCP-2)

The IDCP-2 is a self-report tool used to measure pathological traits (a technical manual in Brazilian Portuguese is currently under development).^24^ The scale is based on pathological traits from Millon,^25^ axis II from the Diagnostic and Statistical Manual of Mental Disorders, 4th edition, Text Revision (DSM-IV-TR),^26^ and PD chapters from DSM-5.^18^ It comprises 12 dimensions, to a total of 47 factors. In this study, according to the hypotheses, we administered the following factors: self-devaluation (7 items), abandonment avoidance (6 items), vulnerability (6 items), anxious worry (6 items), hopelessness (4 items), impulsiveness (6 items), risk taking (6 items), interpersonal detachment (3 items), eccentric style (3 items), paranormality (3 items), persecutoriness (3 items), depersonalization (3 items), emotional inexpressiveness (3 items), distrust in relationships (4 items), deceitfulness of others (3 items), intimacy avoidance (4 items), and emotional apathy (4 items). Psychometric properties of the scale were previously investigated.^27 - 33^ Internal consistency reliability in the present study was > 0.70 for almost all factors, except for emotional inexpressiveness factor (0.65) and deceitfulness of others (0.67).

## Procedure

Data collection followed ethical procedures and was approved by the research ethics committee of Universidade São Francisco (CAAE: 97939518.0.0000.5514). Participants were invited to participate through online social media (e.g., Facebook) and had to give their consent to participate in the study via a Google Forms link before starting to answer the instruments.

Data analysis was conducted using the Statistical Package for the Social Sciences (SPSS) version 25. Associations between pathological traits and religiosity domains were investigated using Pearson’s correlation analysis. For regression analysis, pathological traits were the predictors and religiosity domains were the dependent variables. For each dependent variable we tested two regression models: first, not controlling for sociodemographic variables, and second, controlling for age, sex, educational level, and religion (i.e., “what is your religion?”). The unique contribution of each independent variable was considered significant when p ≤ 0.5.

## Results

Correlations are presented in Tables 1 and 2 , showing associations between religiosity domains and BPD traits ( Table 1 ) and SZPD traits ( Table 2 ).

**Table 1:** - Correlation analysis between BPD traits and religiosity domains

	GR	Forgiveness	HI	Connectedness	HT	ESM	Avoidance	Anxiety
Self-devaluation	**-0.16** *	**-0.30** *	**-0.48** *	0.00	**-0.29** *	0.02	**0.15** *	**0.23** *
Abandonment avoidance	**-0.11** *	**-0.23** *	**-0.22** *	0.04	**-0.42** *	**0.12** *	**0.12** *	**0.24** *
Vulnerability	**-0.12** *	**-0.34** *	**-0.34** *	0.10*	**-0.29** *	0.03	**0.12** *	**0.21** *
Anxious worry	-0.08^†^	**-0.28** *	**-0.33** *	0.03	**-0.42** *	0.09^†^	**0.11** *	**0.26** *
Hopelessness	**-0.28** *	**-0.37** *	**-0.56** *	-0.05	**-0.16** *	**-0.12** *	0.10*	**0.14** *
Impulsiveness	-0.07^†^	**-0.31** *	**-0.23** *	0.09^†^	**-0.17** *	-0.01	0.07^†^	**0.16** *
Risk taking	**-0.14** *	**-0.24** *	-0.08^†^	**0.12** *	-0.04	-0.02	0.02	0.01
Mr (SDr)	0.18 (0.13)							

Bold type indicates r > 0.10 (Cohen, 1988) and significance (p ≤ 0.05): * significant at the 0.01 level; ^†^ significant at the 0.05 level.

Avoidance = intimacy avoidance; Anxiety = abandonment anxiety; BPD = borderline personality disorder; ESM = experience of sense and meaning; GR = general religiosity; HI = hope immanent; HT = hope transcendent; Mr = mean of correlations; SDr = standard deviation of correlations.

**Table 2:** Correlation analysis between SZPD traits and religiosity domains

	GR	Forgiveness	HI	Connectedness	HT	ESM	Avoidance	Anxiety
Interpersonal detachment	**-0.21** *	**-0.26** *	**-0.38** *	-0.06	**-0.13** *	-0.05	**0.12** *	**0.15** *
Eccentric style	**-0.21** *	**-0.30** *	**-0.27** *	0.04	-0.10*	-0.03	0.10*	0.05
Paranormality	**0.27** *	-0.04	0.10*	**0.63** *	-0.02	**-0.23** *	**0.16** *	0.09^†^
Persecutoriness	0.01	**-0.30** *	**-0.16** *	**0.25** *	**-0.22** *	0.04	**0.16** *	**0.22** *
Depersonalization	-0.06	**-0.23** *	**-0.23** *	**0.24** *	**-0.15** *	0.03	**0.13** *	**0.16** *
Emotional inexpressiveness	**-0.23** *	**-0.28** *	**-0.25** *	-0.11*	-0.01	**-0.22** *	0.08^†^	0.05
Distrust in relationships	**-0.16** *	**-0.34** *	**-0.18** *	0.01	**-0.22** *	-0.01	0.09^†^	**0.14** *
Deceitfulness of others	-0.09*	**-0.42** *	**-0.19** *	0.01	**-0.25** *	-0.08^†^	**0.12** *	**0.22** *
Intimacy avoidance	**-0.23** *	**-0.32** *	**-0.33** *	-0.07	-0.09^†^	**-0.13** *	0.10*	0.09*
Emotional apathy	**-0.24** *	**-0.26** *	**-0.37** *	-0.09^†^	-0.07^†^	**-0.22** *	0.08^†^	0.06
Mr (SDr)	0.16 (0.11)							

Bold type indicates r > 0.10 (Cohen, 1988) and significance (p ≤ 0.05): * significant at the 0.01 level; ^†^ significant at the 0.05 level.

Avoidance = intimacy avoidance; Anxiety = abandonment anxiety; ESM = experience of sense and meaning; GR = general religiosity; HI = hope immanent; HT = hope transcendent; Mr = mean of correlations; SDr = standard deviation of correlations; SZPD = schizotypal personality disorder.

Correlations with BPD traits ranged from weak to moderate. Positive correlations, although weak, were observed mainly for AGI factors (i.e., intimacy avoidance and abandonment anxiety). Higher correlations were observed for hope immanent, forgiveness and hope transcendent. Poor correlations were observed for connectedness and experience of sense and meaning.

SZPD traits correlated with religiosity domains, showing weak to moderate results. Positive correlations were observed for AGI factors, but also for connectedness, including the highest correlation observed with the paranormality factor. In general, correlations were higher for forgiveness. The correlation mean was slightly higher for BPD traits in comparison to SZPD traits, although the experience of sense and meaning factor presented higher (negative) correlations with SZPD traits.

Regression analysis results are presented in Table 3 (BPD traits) and Table 4 (SZPD traits).

**Table 3:** Regression analyses with BPD traits as predictors of religiosity domains

	Beta	*t*	p	r^2^
General religiosity				
Anxious worry	0.11 (.12)	1.94 (2.58)	0.05 (0.01)	**0.10 (0.36)**
Hopelessness	-0.34 (-0.29)	-6.69 (-6.91)	< 0.01 (< 0.01)	
Impulsiveness	0.10 (0.09)	1.95 (2.14)	0.05 (0.03)	
Risk taking	-0.14 (-0.14)	-3.09 (-3.56)	< 0.01 (< 0.01)	
Age	0.12	3.69	< 0.01	
Sex	-0.17	-5.73	< 0.01	
Religion	0.45	15.41	< 0.01	
Forgiveness				
Hopelessness	-0.23 (0.23)	-5.27 (-5.05)	< 0.01 (< 0.01)	**0.17 (0.20)**
Impulsiveness	-0.12 (0.11)	2.41 (-2.33)	0.02 (0.02)	
Religion	-0.11	-3.21	< 0.01	
Hope immanent				
Hopelessness	-0.47 (-0.47)	-11.36 (-11.23)	< 0.01 (< 0.01)	**0.35 (0.36)**
Abandonment avoidance	0.15 (0.15)	3.49 (3.45)	0.01 (0.01)	
Self-devaluation	-0.25 (-0.25)	-5.18 (-5.20)	< 0.01 (< 0.01)	
Risk taking	0.09 (0.09)	2.37 (2.38)	0.02 (0.02)	
Religion	0.10	3.57	< 0.01	
Connectedness				
Vulnerability	0.16 (0.13)	3.07 (2.74)	< 0.01 (0.03)	**0.03 (0.30)**
Hopelessness	-0.17 (-0.13)	-3.40 (2.92)	< 0.01 (< 0.01)	
Risk taking	0.10 (0.10)	2.28 (2.73)	0.02 (< 0.01)	
Sex	-0.12	-4.01	< 0.01	
Age	0.14	4.05	< 0.01	
Religion	0.46	15.11	< 0.01	
Hope transcendent				
Anxious worry	-0.27 (0.27)	-5.35 (-5.21)	< 0.01 (< 0.01)	**0.22 (0.23)**
Abandonment avoidance	-0.25 (0.26)	-5.22 (-5.48)	< 0.01 (< 0.01)	
Hopelessness	0.14 (0.14)	2.97 (3.06)	< 0.01 (< 0.01)	
Religion	-0.07	2.23	0.02	

BPD = borderline personality disorder.

Bold type indicates significant prediction.

**Table 4:** - Regression analyses with SZPD traits as predictors of religiosity domains

	Beta	*t*	p	r^2^
General religiosity				
Eccentric style	-0.17 (-0.13)	-4.28 (-3.37)	< 0.01 (< 0.01)	**0.19 (0.33)**
Paranormality	0.30 (0.10)	8.31 (2.77)	< 0.01 (0.01)	
Distrust in relationships	-0.10 (-0.05)	-2.37 (-1.46)	0.02 (0.14)	
Emotional inexpressiveness	-0.08 (-0.03)	-2.09 (-0.52)	0.04 (0.40)	
Intimacy avoidance	-0.10 (-0.11)	-2.05 (-2.45)	0.04 (0.01)	
Emotional apathy	-0.09 (-0.09)	-2.11 (2.20)	0.03 (0.01)	
Sex	-0.12	-3.70	< 0.01	
Age	0.12	3.53	< 0.01	
Religion	0.39	11.69	< 0.01	
Forgiveness				
Eccentric style	-0.14 (-0.11)	-3.47(-2.60)	< 0.01 (0.01)	**0.21 (0.22)**
Deceitfulness of others	-0.24 (-0.24)	-5.13 (-5.19)	< 0.01 (< 0.01)	
Emotional inexpressiveness	-0.11 (-0.10)	-2.81 (-2.52)	< 0.01 (0.01)	
Hope immanent				
Interpersonal detachment	-0.22 (-0.21)	-4.95 (-4.65)	< 0.01 (< 0.01)	**0.22 (0.22)**
Paranormality	0.18 (0.17)	5.02 (4.23)	< 0.01 (< 0.01)	
Depersonalization	-0.12 (-0.10)	-2.86 (-2.48)	< 0.01 (0.01)	
Emotional apathy	-0.20 (-0.21)	-4.72 (4.98)	< 0.01 (< 0.01)	
Connectedness				
Paranormality	0.59 (0.45)	19.48 (14.26)	< 0.01 (< 0.01)	**0.43 (0.51)**
Persecutoriness	0.09 (0.08)	2.51 (2.29)	0.01 (< 0.01)	
Depersonalization	0.10 (0.12)	2.81 (3.81)	< 0.01 (< 0.01)	
Emotional inexpressiveness	-0.10 (-0.05)	-2.99 (-1.74)	< 0.01 (0.08)	
Sex	-0.07	-2.59	0.01	
Age	0.10	3.34	< 0.01	
Religion	0.27	9.29	< 0.01	
Hope transcendent				
Paranormality	0.13 (.11)	3.31 (2.65)	< 0.01 (< 0.01)	**0.10 (0.11)**
Persecutoriness	-0.15 (-0.15)	-3.10 (-3.21)	< 0.01 (< 0.01)	
Distrust in relationships	-0.11 (-0.09)	-2.33 (-1.92)	0.02 (0.06)	
Emotional inexpressiveness	0.10 (0.09)	2.40 (2.16)	0.02 (0.03)	
Deceitfulness of others	-0.14 (-0.14)	-2.73 (-2.75)	< 0.01 (< 0.01)	
Experience of sense and meaning				
Paranormality	0.22 (0.18)	5.96 (4.31)	< 0.01 (< 0.01)	**0.12 (0.13)**
Distrust in relationships	0.12 (0.13)	2.61 (2.92)	< 0.01 (< 0.01)	
Emotional inexpressiveness	-0.17 (-0.16)	-4.17 (-3.72)	< 0.01 (< 0.01)	
Emotional apathy	-0.015 (-0.15)	-3.26 (-3.35)	< 0.01 (< 0.01)	
Intimacy avoidance				
Paranormality	0.12 (0.06)	2.95 (1.30)	< 0.01 (0.19)	**.03 (.05)**
Sex	-0.11	-2.80	< 0.01	
Religion	0.10	2.41	< 0.01	
Abandonment anxiety				
Interpersonal detachment	.16 (0.14)	3.21 (2.94)	< 0.01 (< 0.01)	**0.07 (0.11)**
Eccentric style	-0.12 (-0.08)	-2.71 (-1.93)	< 0.01 (0.05)	
Persecutoriness	0.15 (0.14)	3.07 (2.96)	< 0.01 (< 0.01)	
Deceitfulness of others	0.17 (0.17)	3.43(3.36)	< 0.01 (< 0.01)	
Sex	-0.14	-3.85	< 0.01	
Religion	0.17	4.48	< 0.01	

SZPD = schizotypal personality disorder.

Values between brackets indicate control for sociodemographic variables.

Bold type indicates significant prediction.

BPD traits were significant predictors for all religiosity domains, except for intimacy avoidance, in which only sociodemographic variables presented unique contributions to the model. Hopelessness was the factor with significant contributions to the largest number of regression models. After controlling for sociodemographic variables, vulnerability lost significance in some models. In regression models where sociodemographic variables were not controlled for, hope immanent and hope transcendent were the religiosity domains with the highest variance explained by pathological traits. When adding sociodemographic variables to the models, general religiosity and hope immanent were the domains best explained by traits.

At least one SZPD trait was a significant predictor for each religiosity domain. The paranormality factor was the best predictor, appearing in almost all regression models, always with a significant positive contribution. When adding sociodemographic variables to the model, emotional inexpressiveness, distrust in relationships and paranormality were no longer significant predictors. The religiosity domains best explained by pathological traits were connectedness and hope immanent, while general religiosity and connectedness were best explained after inclusion of sociodemographic variables.

## Discussion

Even though religiosity domains have been linked to mental health outcomes,^1 - 3 , 6 , 7^ little knowledge has been generated to date on the relationship with pathological traits that comprise PDs. In this study, we aimed to extend evidence on this relationship, investigating associations between religiosity and pathological traits from two specific PDs, i.e., BPD and SZPD. Overall, we found associations between traits and religiosity domains, as detailed in the next paragraphs.

In our first hypothesis, BPD traits should present higher associations with religiosity domains in comparison to SZPD traits, although traits from both PDs should have significant correlations. As expected,^19 , 21^ associations with BPD traits were higher than associations with SZPD – however, only slightly higher. In light of these findings, we can hardly conclude that BPD traits are more associated with religiosity constructs than SZPD traits. Moreover, significant associations were mostly negative, or positive when the religiosity domain was representative of impairments (i.e., the intimacy avoidance and abandonment anxiety factors of the AGI). Evidence of this study suggests higher religiosity as associated, in general, with lower levels of pathological traits.

The connectedness and hope transcendent domains were the ones to present distinct correlation patterns between BPD traits and SZPD traits. The association pattern observed suggests that our second hypothesis was partially corroborated, confirming previous evidence.^4 , 9 , 11^ Specifically, connectedness showed higher association with paranormality, and less evident associations with persecutoriness and depersonalization. In contrast, only poor associations were observed for the connectedness domain with BPD traits. These results confirm our second hypothesis, as connectedness is a spiritual-related religiosity domain.^34^ Conversely, the hope transcendent domain presented higher negative associations with BPD traits, despite being considered as spiritual-related. Looking at the content of each item comprising the hope transcendent domain, all of them are related to lack of fear of abandonment (e.g., “It is hard for me to think that my loved ones will one day no longer live.”) or to the lacking anxiety regarding the future and specifically related to the afterlife (e.g., “I would do anything to prolong my life.”). Not by chance, higher negative associations with hope transcendent were observed for the abandonment avoidance and anxious worry factors of the IDCP-2, related to fear of abandonment and anxiousness regarding the future, respectively.^30 , 32^

Considering our expectations, inverted associations were observed between the experiences of sense and meaning factor and SZPD traits, contradicting our second hypothesis. One possible explanation could be regarding the content of the items, i.e., mainly related to friendship (i.e., close relationships) and emotions, which are impaired domains in people with SZPD pattern.^18 , 20^

Regression analysis complemented what was observed through correlation analysis for our second hypothesis: SZPD traits showed higher explanatory capacity especially for the connectedness domain – a spiritual-related domain.^11^ Moreover, in the regression model, paranormality showed the highest contribution, which is in accordance with spiritual beliefs.^28^ Furthermore, and not hypothesized in this study, the hope immanent and hope transcendent domains were best explained by BPD traits. As we enlightened before, hope transcendent is related to lacking fear of abandonment and to absence of anxiety related to the afterlife,^34^ whereas hope immanent regards optimism with the future. Our findings suggest that people characterized by fear of abandonment and risk taking – from BPD –, but not hopelessness and self-devaluation, tend to present higher scores in the hope immanent domain. Future studies should focus on comparing people with these two dissimilar BPD trait profiles regarding hope immanent scores.

Although most associations observed in this study were negative, in the regression models the paranormality factor of SZPD showed significant and positive contributions to almost all models. Again, this finding corroborates our second hypothesis,^4 , 9^ as this IDCP-2 factor comprises items related to beliefs in supernatural experiences and phenomena. Moreover, the insecure religious attachment, assumed in our second hypothesis as more related to SZPD traits, in fact showed poor association with PD traits in general, except for the abandonment anxiety factor, which was associated with most of BPD traits. This finding is consistent with the internalization component of BPD.^35^

Our two hypotheses were partially corroborated, i.e., BPD traits showed only slightly higher associations with religiosity domains in comparison to SZPD traits, and most of the spiritual-related domains showed higher associations with SZPD traits than with BPD traits. These findings indicate that higher levels of religiosity domains are associated with the presence of less pathological traits. In other words, in our study, religiosity played a protective role against pathological traits. Nevertheless, we did not control for the level of religiosity in the sample, so it is possible that the relationship observed could change specifically at the extremes of religiosity. Future studies should examine this possibility. Furthermore, even though we controlled for the participant’s religion and observed this variable as significantly contributing to the regression model, future studies should further investigate the role of specific religions in the relationship between pathological traits and religiosity domains.

Findings from this study should be interpreted considering some limitations. First, our investigation focused on traits from two specific PDs; second, our sample did not include patients diagnosed with PDs; third, the PD trait scale is a self-report instrument, not a diagnostic assessment tool; and fourth, even though the religiosity scales administered covered several domains, we did not use specific scales for spirituality.
